# Understanding the use of telehealth in the context of the Family Nurse Partnership and other early years home visiting programmes: A rapid review

**DOI:** 10.1177/20552076221123711

**Published:** 2022-11-14

**Authors:** Kathleen Morrison, Thomas Hughes, Lawrence Doi

**Affiliations:** 1 Scottish Collaboration for Public Health Research and Policy, School of Health in Social Science, 65932 University of Edinburgh, Edinburgh, UK

**Keywords:** Telehealth, telemedicine, e-Health, digital health, maternal–child health services, Family Nurse Partnership, home visiting, adolescent mothers, early parenthood

## Abstract

**Overview:**

This rapid review sought to understand the use of telehealth in early parenthood programmes sharing similarities with the Family Nurse Partnership.

**Methods:**

A rapid review protocol was developed in accordance with Cochrane Rapid Reviews Methods Guidance. Medline, Cochrane Library, and CINAHL databases were searched. Inclusion criteria were developed using population, intervention, comparator, outcome, study design, and timeframe components. Two reviewers searched, screened, and extracted data. AMSTAR was used for critical appraisal. Results were synthesised narratively.

**Results:**

Searches yielded 18 studies out of 881 for inclusion. Findings were identified across seven domains: *acceptability and accessibility*; *therapeutic relationships*; *flexibility offered by telehealth*; *participation and engagement*; *confidentiality and privacy*; *equipment and technical considerations*; and *training and support.*

**Conclusion:**

Telehealth provides unique opportunities to improve access to early years health services for young mothers. However, considerable accessibility barriers remain in the form of connectivity issues, access to appropriate technology, and the acceptability of remote healthcare delivery. This review presents a timely overview of the opportunities and challenges associated with the use of telehealth in early parenthood and family-based programmes.

## Introduction

Telehealth technologies have the potential to greatly expand the reach of healthcare services and provide access to service users where this might otherwise be unavailable. Telehealth refers to the remote provision of healthcare through a variety of telecommunication tools, such as telephones, smartphones, and mobile wireless devices, with or without video.^1^ Telehealth has often been explored in the context of remote and rural service delivery, but the field has recently been subject to enhanced focus and transformation during the global coronavirus disease 2019 (COVID-19) pandemic which has brought fundamental changes in the way that healthcare services are delivered.

In the context of COVID-19, many changes have been implemented at an unprecedented pace and scale to ensure the provision of continuous care and essential services. Such an example includes the rapid digital transformation of complex maternal and child health home visiting programmes, such as the Family Nurse Partnership (FNP), to incorporate the widespread use of telehealth to enable safe and continuous service provision during the pandemic.

A rapidly growing evidence base has charted the use of telehealth in relation to a variety of clinical conditions and health outcomes prior to and during the COVID-19 pandemic. Most available evidence relates to the use of telehealth in the treatment and management of conditions such as cardiovascular disease, diabetes, mental health conditions (e.g. depression and anxiety), smoking cessation and substance abuse support, physical rehabilitation, post-operative care, and various chronic conditions such as hypertension, chronic-obstructive pulmonary disorder, and kidney failure.^1–3^ In comparison, less is known about the long-term impacts of telehealth being incorporated more widely into specialist home visiting programmes. Following an extensive review, Totten et al.^3^ identified maternal health as one of the three main areas where telehealth use was deemed to be appropriate but significantly under represented by research evidence. More recent studies have also noted a trend of limited telehealth evidence for home visiting programmes and have highlighted a need to demonstrate the use and impact of telehealth technologies in these contexts.^4,5^

The purpose of this review was to inform the use of telehealth in FNP service delivery in Scotland during COVID-19. In the absence of FNP-specific evidence, the review draws on relevant findings from intervention types and population groups sharing similarities with key FNP programme components, service user populations, and practitioner characteristics.

The FNP is specifically tailored to provide support during early parenthood to improve outcomes for young first-time mothers and their children. The programme supports young mothers to engage in preventative health practices, responsive caregiving, positive parenting practices, and the development of self-efficacy to form and achieve goals for the future and improve economic self-sufficiency.^6^

The license-based FNP was originally developed through a body of research that began in 1977 and incorporated three US-based randomised trials.^7–10^ The FNP is an intensive, one-to-one programme delivered by specially trained nurses in a home visiting format and is designed to support young first-time mothers and their children.^11^ Following its inception in the US, the FNP has been trialled and adapted internationally. The programme has been adopted by three of the four UK nations (excluding Wales) and is run at a national level within each of these healthcare systems. FNP shares several commonalities with UK-based health visiting services, in that it targets new mothers and pre-school children and upholds the principles of early intervention and prevention.

Evidence shows that early parenthood is associated with social disadvantage, as well as poor health and well-being outcomes for both the mother and the child.^12,13^ Young first-time mothers are a vulnerable population group with varied and complex needs that can be wide ranging across domains such as social issues, health, housing, and welfare.^6,14^ Analysis of pre-enrolment data of clients’ experiences prior to enrolment in FNP in Scotland showed that 98% of FNP clients experienced a form of trauma or adverse experience prior to entering the programme.^6^ Amongst a range of complexities and challenges recorded in clients’ lives, 88% of clients were estimated to have at least one socioeconomic disadvantage, such as having a low income (60%), poor job stability (10%), and not being in education, training or employment at the time of enrolment (67%). The analysis also showed that 28% of clients had experienced homelessness and 28% were recorded as living in poor or unsuitable housing upon enrolment. Additionally, family nurses recorded that 75% of clients had health issues when entering into the programme, with 63% experiencing mental health issues, namely anxiety disorders.

During the onset of the COVID-19 outbreak, guidance published by the Scottish Government in April 2020 stated that the FNP programme ‘provides an essential service to the clients and children enrolled on the programme. Families will continue to need the support of FNP and, in fact, will likely need their connection to their FNP nurse more than ever’.^15^ Due to the essential nature of FNP and transformations taking place to ensure the delivery of care in response to COVID-19 this review sought to gain an understanding of telehealth implementation in this context and any associated impacts.

The aim of the rapid review was to summarise a range of evidence relating to the impacts of telehealth and the implications of this in the context of FNP and other similar home visiting programmes. In this review, early years home visiting interventions refer to specialist programmes designed to support parents and their children with a range of social and health-related needs that primarily feature an in-home delivery format between families and a trained practitioner. Alongside the targeted FNP model, similar home visiting programmes include universal health visiting services delivered across the four UK nations and the large-scale US-based Maternal, Infant, and Early Childhood Home Visiting programme.^16–19^

## Methods

The review protocol follows the Cochrane Rapid Reviews Methods Group Interim Guidance,^20^ which defines a rapid review as ‘a form of knowledge synthesis that accelerates the process of conducting a traditional systematic review through streamlining or omitting specific methods to produce evidence for stakeholders in a resource-efficient manner’.^21^ The review was designed to provide stakeholders and decision-makers in Scotland with timely results to support changes to FNP service delivery in response to the COVID-19 outbreak.

The research question was developed in consultation with key stakeholders representing the FNP National Clinical Team and policy officers in the Scottish Government. The review question, search strategy, and eligibility criteria were subsequently refined and agreed upon following the input of the above stakeholders.

Priority was given to the inclusion of review-based study designs (e.g. systematic reviews, scoping reviews, etc.) to provide the highest level of available evidence.^20^ To increase relevance the review team agreed to conduct limited and targeted searches to include recent primary studies (from 2020 to 2021) and grey literature holding direct relevance to the intervention of study and/or the emerging context of COVID-19.

### Research question

What are the potential impacts of telehealth service delivery in the context of early parenthood programmes such as the FNP?

### Eligibility criteria

Population, intervention, comparator, outcome, study design and timeframe/other limits (PICOST) eligibility criteria were established a priori.

#### **P**opulation

Healthcare practitioners delivering a programme of care using modes of telehealth and/or relevant client groups receiving care via telehealth were eligible. Client population groups relating to young females, mothers, parents of young children, and families were of particular interest. Less relevant population groups were excluded, such as older or predominantly male groups as well as studies focussing primarily on highly specific clinical populations or the treatment of specific chronic conditions (e.g. diabetes, cancer, chronic pain, etc.).

#### **I**ntervention

Telehealth interventions delivered by healthcare practitioners to service users/clients that hold contextual relevance to the FNP programme were included. Examples of eligible interventions with relevance to the FNP included home visiting programmes, parenting programmes, child and family-based health promotion interventions, pre- and post-natal care, sexual and reproductive health interventions, and mental health interventions. Interventions had to include at least one telehealth component such as telephone calls, SMS, video-conference calls, use of specialist video consultation software (e.g. NearMe and Attend Anywhere), emails, or another online web-based mode (e.g. WhatsApp messaging). Interventions also had to include continuous or ongoing telehealth interactions and as such, studies with singular or one-off interactions were excluded. Service user/client–practitioner and practitioner–practitioner interactions via telehealth were all eligible for inclusion.

#### **C**omparator

Studies with and without comparator groups were eligible for inclusion.

#### **O**utcomes

Outcomes relating to the following areas were considered:

- Continuity of care and carer
- Impact of telehealth on client–practitioner relationships
- Practitioner and client satisfaction
- Acceptance
- Accessibility
- Engagement
- Modes of delivery (e.g. telephone, video call, and SMS)
- Geographical considerations
- Training and education
- Impact of telehealth on clinical observations

#### **S**tudy design

Reviews (such as systematic reviews, qualitative evidence syntheses, and scoping reviews), primary studies and grey literature holding direct relevance to the intervention of study and/or the emerging context of COVID-19 were eligible for inclusion.

#### **T**imeframe and other limits

Abstracts must include one or more terms relating to the intervention and outcomes/areas of interest. Only reviews published in the English language from 2005 to 2021 were eligible. Additionally, only studies with relevance to high-income settings were eligible for inclusion. Studies relating only to low- and middle-income countries were excluded.

#### Search strategy

Databases searched included Medline, the Cochrane Library, and CINAHL. All searches were conducted between 26 August 2020 and 21 September 2021.

Search terms were developed based on the PICOST formulation for population, intervention, outcomes, and study design, as outlined in Table 1. Search terms for each domain were combined using the Boolean operator ‘OR’ and then each domain category was combined using the ‘AND’ function (please see Supplementary file 1 for full search strategy). The search was limited to sources published from 2005 onwards. The same search strategy was used for all databases.

**Table 1.** Thematic domains versus corresponding evidence sources.

Source	Ames et al.^29^	Bailey et al.^45^	Brewster et al.^43^	Cunningham et al.^28^	Endler et al.^33^	Gentles et al.^30^	Gonçalves- Bradley et al.^47^	Hanach et al.^38^	Harris et al.^42^	Lattie et al.^35^	Lavender et al.^36^	LeBlanc et al.^31^	Nordtug et al.^44^	Odendaal et al.^40^	Poorman et al.^26^	Seko et al.^24^	Verhoeks et al.^41^	Walsh et al.^46^	Total no. of sources
**Thermal domain**																			
1. Accessibility & acceptability	Δ			•	Δ	•		Δ		Δ	♦	•			•	•			10
2. Relationships with healthcare providers	Δ		•			•			•					Δ			Δ		6
3. Flexibility offered by telehealth		•			Δ	•						•	•	Δ		•	Δ		8
4. Participation & engagement							♦	Δ	•	Δ		•		Δ	•		Δ	•	9
5. Confidentiality & privacy	Δ	•											•	Δ		•			5
6. Equipment & technical factors			•									•	•			•			4
7. Access to training & support			•	•								•	•			•			5

sources: AMSTAR quality scoring: •  = low; Δ = moderate; ♦ = high.

#### Study selection

All results were screened based on the eligibility criteria, using *Covidence* software*.* Two reviewers (KM and TH) blind screened papers independently at all stages of the screening process with disagreements being resolved by further discussion with all authors.

#### Title and abstract screening

Two reviewers (KM and TH) conducted title and abstract screening based on the inclusion of key terms in the title or abstract and relevancy to the topic. Once both reviewers had completed the initial screening, conflicts were resolved through discussion with all reviewers, and sources were moved to full-text screening.

#### Full-text screening

Full-text articles were also screened by two reviewers (KM and TH). One reviewer (KM) also screened all excluded full-text articles before data extraction occurred.

#### Data extraction

Data extraction was undertaken and cross-checked by two reviewers (KM and TH). A predefined data extraction form was used to gather information regarding the purpose of each study, the populations, interventions, outcomes, results, and risk of bias and any limitations were documented. One reviewer (KM) checked the extracted data for correctness and completeness.

#### Quality assessment

Quality assessment was conducted using the AMSTAR appraisal tool for systematic reviews.^22^ AMSTAR is frequently used in rapid reviews as it allows for the assessment of both qualitative and quantitative study designs as well as data heterogeneity. One reviewer (TH) assessed the quality of each review using the AMSTAR tool. A second reviewer (KM) then verified and cross-checked results.

#### Supplementary sources

Supplementary sources were identified via limited and targeted searching of key databases and Google Scholar to include recent (<1 year from September 2020) primary studies holding direct relevance to the research questions and emerging context of COVID-19. Key references and FNP programme documents were also provided by the stakeholders to enhance the relevancy of the review.

Quality assessment for supplementary sources was limited within the constraints of a rapid review. The risk of bias and quality assessment was based on validity, self-reported limitations by study authors, and judgements made by the reviewers.

#### Data synthesis

All evidence was thematically categorised and synthesised narratively. Our goal was to find commonalities across studies and group these into themes of interest based on the aim of the review.

## Results

### Overview

**Figure 1.**
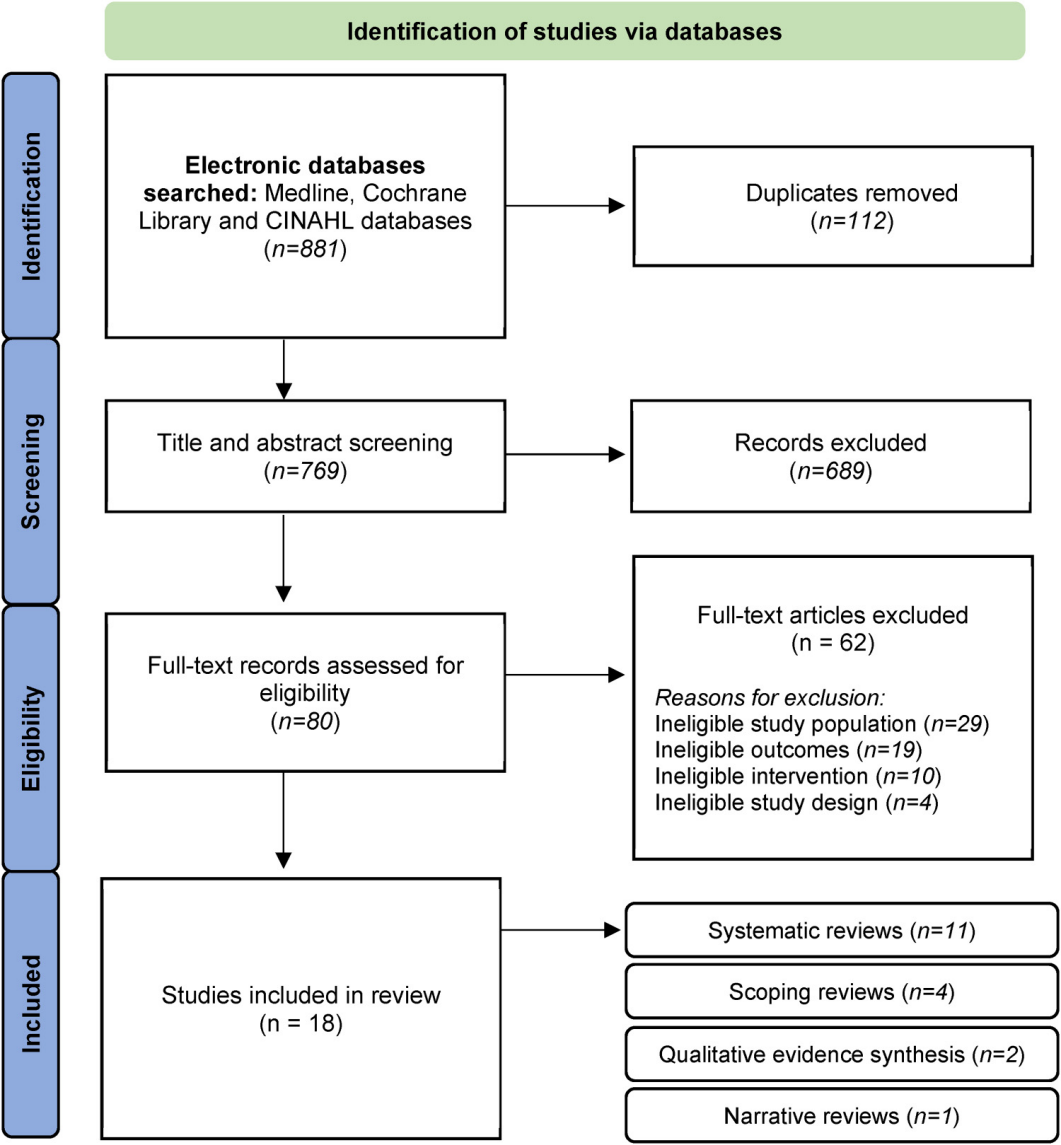
Preferred reporting items for systematic reviews and meta-analyses (PRISMA) flow diagram.^23^

A total of 881 records were identified from database searching and 18 studies fulfilled all inclusion criteria. The review findings are summarised and presented in Supplementary file 2. The identification, eligibility assessment, and exclusion of sources are illustrated in Figure 1.

Included review types were classified as systematic reviews (*n* = 11), scoping reviews (*n* = 4), qualitative evidence syntheses (*n* = 2), and narrative review (*n* = 1). With regard to the study quality of the reviews included, two were deemed high quality, six were of a moderate quality, and 10 were considered low quality.

Six supplementary sources were identified for inclusion. These findings are summarised and presented in Supplementary file 3.

Seven thematic categories were identified from the review literature. An overview of domains and corresponding evidence sources are illustrated in Table 1. Full findings are available in Supplementary file 2 and presented narratively below.

#### Accessibility and acceptability

##### Accessibility

Ten reviews and five supplementary sources suggest that telehealth can facilitate greater access to healthcare services among a range of population groups. Factors such as the large-scale ownership of mobile phones among young people can present opportunities for extending outreach services and mitigating barriers to accessing healthcare.^24,25^ Interventions such as text messaging and videoconferencing were found to be effective in improving access to services for families and first-time mothers, particularly those with fewer resources.^26–28^ The accessibility of mobile phone interventions was also favourable because of their user-friendly nature.^24^

Digital access to healthcare services expanded opportunities for clients with caring or employment responsibilities and for service users living in remote areas.^29–31^ Additionally, a primary study conducted during the COVID-19 pandemic pointed to the benefits of telehealth when engaging socially isolated mothers, mothers suffering from mental health problems, and mothers whose first language is not English.^32^ Telehealth service delivery contributed to fewer cancellations and delays for service users in comparison with face-to-face delivery in comparable settings.^33^

Accessibility challenges were identified for users with low literacy or digital literacy skills.^34^ Difficulties were also noted for users relying on shared access to digital devices.^29^ Access was also seen to be dependent on whether health insurance companies were willing to cover telehealth services, and where there was relatively relaxed legislation around using technologies.^28^

Network connectivity issues, lack of device ownership, poor device usability, and issues relating to data confidentiality and privacy were regarded as the main accessibility barriers encountered by service users.^28,29,31^ This is also supported by recent findings relating to telehealth service delivery during the COVID-19 pandemic which suggest that the key factors limiting the usability of telehealth are connection issues and limited access to technology.^25^

##### Acceptability

Acceptability outcomes for telehealth interventions were varied. One systematic review found that usability and acceptability outcomes for telehealth interactions were normally favourable.^35^

Familiarity with mobile devices among client and practitioner groups was identified as increasing the usability and acceptability of telehealth interventions. Typically, this required less time to be spent on training and support around the use of the technologies for both service users and practitioners.^24,26^ However, the acceptability of telehealth via mobile devices was found to be dependent on cost. Some service users faced barriers such as lacking enough credit to send and receive SMS messages or make phone calls.^29^ Many service users agreed that the interventions should be free or low cost.^28,29^

Three studies reported similar or higher levels of parental and maternal satisfaction with telehealth and online parent programmes in comparison with face-to-face formats.^36–38^

The acceptability and feasibility of telehealth interventions by families of young children were interlinked with socioeconomic status (SES).^34^ One review showed that families from the least deprived socioeconomic backgrounds tended to engage more frequently with a variety of intervention types and were more accepting to telehealth services than families from more deprived areas.^34^ Online discussion forms and blogs were least attractive to lower SES families, while higher SES families tended to engage more with web-based sources for parenting information. Some studies reported that lower SES families indicated preferences for printed information materials delivered by mail over online information sources. Differences in engagement with online sources relating to SES may also be linked with technological access, comfort, prior experience, and trust in online sources. The use of mobile devices and video calls yielded mixed satisfaction, due to technical and hardware issues, but was found to be the most acceptable intervention type across all SES groups. These findings are contrary to Smith et al.,^32^ who argued that telehealth provided opportunities to engage with hard-to-reach families during the COVID-19 pandemic. It should be noted that Smith et al.'s findings may be more reflective of current circumstances where the context of the COVID-19 pandemic necessitated higher engagement with digital communication methods across society. It is also more likely to be indicative of significant improvements in the availability and quality of technological devices and communication software in recent years.

#### Therapeutic relationships

Seven studies examined therapeutic relationships and connections between clients and healthcare practitioners. Therapeutic relationships are an essential aspect of FNP and similar home visiting programmes. It is a trusting relationship built between a client and practitioners that seeks to support learning, emotional development, and positive behaviour change in clients.^39^

Findings concerning the overall impact of telehealth on therapeutic relationships were nuanced. No significant positive or negative associations were evident, yet some studies highlighted the importance of face-to-face contact between clients and providers when establishing and maintaining relationships. Three reviews^29,40,41^ and one primary study^27^ reported that telehealth had a slight degree of positive impact on client–provider relationships by providing opportunities for regular support and connectedness, as well as additional flexibility and convenience about how and when clients were able to engage with a practitioner.^27,29,41^ Practitioners felt that options for mobile phone communication strengthened relationships with clients and provided clients with opportunities to initiate engagement with their practitioners which demonstrated clients being able to take more ownership over their health.^40^ One review found that studies comparing the effectiveness of telehealth with in-person programmes showed greater positive effects for face-to-face interventions, in terms of effecting behaviour change.^42^

One review found that women seeking therapy for depression were initially sceptical about developing therapeutic relationships with healthcare providers using telehealth.^41^ Over time, however, many of these women considered online therapeutic relationships to be comparable with face-to-face delivery, allowing effective working alliances, an important aspect of therapeutic relationships, to form between them and their healthcare providers.^41^ However, some participants felt that therapeutic relationships were less close and personal in the absence of face-to-face contact. One review also reported that telehealth helped to establish continuity of care for service users in the community beyond traditional care settings.^30^

Reduced opportunities for face-to-face contact had a negative impact on job satisfaction among nurses who felt that delivering care via telehealth challenged relationships with clients.^43^ Three reviews reported that it took more time for healthcare providers to build rapport with service users virtually and that telehealth clients experienced more distant and less personal therapeutic relationships overall. In some instances, this led to the provision of additional face-to-face contact to service users.^27,40,41^

One supplementary study argued that mixed-model delivery, utilising telehealth in addition to in-person care, was important and allowed practitioners and service users to experience both good levels of personal engagement and the convenience of telehealth.^27^ Women were reported to prefer the use of telehealth in blended, or hybrid, programme models, viewing telehealth as complementary to in-person care rather than as a standalone method, compared with men.^41,42^

#### Flexibility offered by telehealth

Eight reviews emphasised benefits relating to the flexibility conferred by telehealth in healthcare settings and for individual service users. Odendaal et al.^40^ reported that telehealth allowed healthcare workers to take up new tasks, work flexibly, and effectively reach clients in hard-to-reach areas. Telehealth was appreciated when it improved factors such as feedback, speed, workflow, travel time, and the efficiency of care.^30,31,33,44^ However, some studies reported that using telehealth contributed to additional workloads overall.^45^

Young people tended to prefer SMS (text messaging) and software apps over synchronous technologies (e.g. calls and video calls) as they granted more autonomy and allowed them to engage with services in their own time, which could feel less intrusive.^24^ Similarly, female users were attracted to the flexibility offered by telehealth allowing them to control their engagement with healthcare more widely at a time, pace, and setting of their choosing.^41^ Unlike in-home visits, service users felt less pressure to prepare their homes for visitors prior to arrival, eliminating some of the stress associated with this.^44^ Telehealth also provided convenience to mothers who faced challenges accessing in-person where barriers such as transportation, location, or weather conditions impeded their ability to travel.^32^

In contrast, Verhoeks et al.^41^ identified that some women viewed the flexibility offered by telehealth as a barrier to engagement as it created a reduced sense of obligation and motivation to attend appointments compared to face-to-face appointments. Nordtug et al.^44^ discussed the importance of remote contacts being scheduled in advance and clearly identifying who should initiate contact, when, how often, and where, to encourage engagement and appointment attendance.

#### Participation and engagement

Nine reviews and one supplementary source presented findings relating to participation and engagement. Traube et al.'s^37^ pilot feasibility study explored the delivery of home-based parenting programmes via telehealth and identified that the most common reasons for enrolling in the online programme were convenience, privacy, and word of mouth.

Two studies identified an association between the duration of an intervention and participation. Shorter interventions were associated with greater positive outcomes for service users and their children compared to longer interventions requiring prolonged commitment which created significant obstacles for socially disadvantaged families who are more likely to be experiencing additional stressors and time constraints.^37,42^

Telehealth programmes tailored to meet individual's needs were found to enhance factors such as intrinsic motivation and engagement. A systematic review exploring women's expectations and experience regarding e-health programmes found that practitioners played a key role in ensuring that programmes felt personalised to individuals highlighting that this was a key determinant towards minimising barriers to engagement and increasing motivation.^41^

Participation and retention rates were also enhanced when telehealth interventions were based on relevant behaviour change theories and when intervention outcomes were aligned closely with the programme content.^26^ While studies suggest that individuals living with mental health conditions reported higher levels of acceptance towards telehealth interventions,^38,46^ higher rates of attrition were also reported within these population groups.^35,46^ In contrast, Hanach et al.^38^ found that completion rates were slightly higher for women suffering from postpartum depression using telehealth compared with those using in-person interventions. These studies indicate that the type or severity of a mental health condition may determine the degree to which an individual is able to participate in telehealth interventions. Both reviews found that these findings were compounded by factors such as a lack of prompting or guidance which could contribute to service users feeling unsupported.

In terms of staff participation and engagement, some healthcare workers appreciated being more connected to colleagues via telehealth, believing that this improved coordination and quality of care for service users.^31,40^

Gonçalves-Bradley et al.^47^ conducted a review of 19 randomised controlled trials that assessed the effectiveness of mobile technologies in the management of care and found that most trials reported a reduction in time between the presentation and management of a health issue when practitioners were communicating with specialists via mobile technologies. This has the potential to facilitate preventative care practices such as early identification, timely referral, and early intervention for parents and children requiring care or support.

#### Confidentiality and privacy

Five reviews explored confidentiality and privacy concerns relating to the use of telehealth. Clients at risk of social stigmatisation or discrimination (e.g. those seeking family planning, abortion care, or HIV treatment) expressed concerns around the maintenance of privacy and confidentiality during telehealth consultations including the consequences of any breaches or disclosures that could occur as a result of receiving digital healthcare.^29^

The use of telehealth was influenced by the environmental setting of both the client and the provider.^44,45^ Busier open settings for telehealth consultations, such as nurses’ stations created concerns among clients regarding privacy and confidentiality.^45^ In contrast, service users with the ability to control their own environment for video calls found that this positively influenced their sense of privacy and confidentiality.^44^

One study found that mobile phone calls provided service users with adequate privacy to support sensitive therapeutic activities.^24^ The use of mobile phones offered users personal space, allowing for an increased sense of autonomy, control, and self-esteem. While recognising the advantages of mobile phone usage, the authors reported that 29% of studies reported confidentiality and privacy to be the most common concern among telehealth users. In particular, the loss of a mobile phone was thought to be a serious privacy concern.

Privacy and confidentiality concerns were also identified as a barrier to acceptability.^29^ Odendaal et al.^40^ noted that healthcare providers had a strong awareness of the importance to protect client confidentiality when using mobile devices.

#### Equipment and technical considerations

Four reviews and one supplementary source presented findings related to equipment and technical considerations. Experiencing technological problems had a negative impact on the acceptance of telehealth by front-line staff.^43^ Limited internet and mobile network connectivity amongst users was a common barrier, particularly in the use of videoconferencing.^24^ Whilst video quality has significantly improved over time, issues such as spoiled video feeds are still deemed to be an issue.^44^ Human interaction and potential for errors are also of relevance here, for example, ensuring a network signal is appropriately on or off.^44^ Addressing resource disparities relating to factors such as inadequate internet speeds and device ownership were viewed as key elements to overcome for equitable telehealth participation.^28,31^

#### Training and support

Findings around training and support were identified within five reviews and one supplementary source. Access to training and support positively influences each of the outcome areas presented in this review. Access to telehealth training and support led to more acceptance and positive attitudes towards the use of telehealth amongst healthcare staff.^43,48^ Owen^27^ also found that peer-led upskilling of clinicians in the use of video calls resulted in increased knowledge, skills, and comfort with using these technologies. Low levels of technological confidence and training in practitioners were identified as a significant barrier to the implementation of telehealth interventions.^24,31^

In terms of service users, one review highlighted that while some interventions are user-friendly, providing service users with information and guidance on the use of a telehealth platform and signposting technological support and assistance positively influences uptake and engagement.^44^

## Discussion

This review presents key factors that can influence the impact of telehealth and lay the landscape for understanding where important barriers and facilitators may lie.

Overall, the findings suggest that telehealth can provide unique opportunities to improve access for young mothers and parents to engage with early years services. The findings also suggest that telehealth interventions can provide improved healthcare access for vulnerable population groups such as young people, pregnant women, first-time mothers, women living with mental health conditions, women with fewer resources, and migrant groups, that share many characteristics with FNP client populations.^6^ This is linked to the large-scale use of devices such as mobile phones and tablets amongst the client age group (24 years and under), user-friendly telehealth technologies, opportunities to overcome geographical barriers, and opportunities for client groups to engage more conveniently and flexibly with services while managing responsibilities relating to caregiving, education, training, and employment.

In accordance with the UK Government digital strategy^49^ and NHS Digital guide,^50^ digital inclusion is subject to overcoming initial barriers relating to access, connectivity, skills, confidence, and motivation before addressing secondary barriers relating to design, awareness, and the capability and/or capacity of healthcare staff. Our findings correlate closely with many of these factors and should be given deliberation in this regard.

Individual and socioeconomic circumstances are influential to the uptake and acceptance of telehealth and should be taken into consideration when introducing telehealth in place of in-person care for young parents and families. Greater acceptance of telehealth is seen when the intervention is tailored towards individuals and feels personal. For healthcare practitioners, acceptance appears to be highest when the intervention is user-friendly and good levels of training, resources, and support are provided from the outset. Considerable accessibility barriers for both clients and practitioners remain in the form of connectivity issues, access to adequate technology, the costs of receiving and sending SMS messages or calls, and low levels of training.

While factors such as high device ownership are typically reported in younger age groups, findings from the COVID-19 pandemic highlighted the pervasive extent of digital exclusion and inequalities experienced by young people and families living in the UK.^51^ Across the UK it is estimated that 22% of the population do not have the necessary essential digital skills required for everyday life.^52^ Aside from age, the population sub-groups most likely to be digitally excluded include lower income and unemployed groups; people living in social housing; people living with disabilities; people with fewer educational qualifications; early school leavers; people living in rural areas; and people whose first language is not English.^50^ In terms of sociodemographic markers, early parenthood is likely to be inter-linked with the sub-groups most at risk of digital exclusion and as such, decision-makers and practitioners should make informed judgements regarding the use of telehealth with their client groups.

Digital exclusion therefore encompasses a breadth of issues that can limit engagement for multiple population groups. This presents profound consequences for some individuals’ abilities to engage with employment, education, and healthcare services. Interventions with central digital engagement components should therefore seek to measure and monitor digital inequalities among various population groups to ensure equitable service provision. Yates^51^ suggested that a key factor in addressing this is to identify citizens who are ‘limited’ users of digital systems and avoid ‘one size fits all’ or ‘technology-led’ approaches if the skills and needs of the target population are not aligned with this.

Where resource disparities or limited digital skills are a factor, programmes such as Connecting Scotland that received large-scale government investment to directly target digital exclusion have demonstrated positive impact by providing devices, support and connectivity options to vulnerable and low-income households during the COVID-19 pandemic.^53^ In terms of healthcare alone, 58% of participants reported an improvement in their ability to access healthcare services after receiving support from Connecting Scotland.^54^ Investments such as these are integral to addressing digital exclusion by providing families with the ability to interact with a range of essential services, including those with telehealth components.

High acceptability rates also appeared to be closely linked with intervention design and were positively influenced by the inclusion of behaviour change theories, which helped to maintain engagement. The engagement was largely determined by factors relating to programme delivery and accessibility where more supportive and hands-on programmes produced higher levels of engagement overall, particularly among female telehealth users. Healthcare practitioners also play a key role in encouraging and sustaining engagement levels.

Technical considerations play a significant role in the delivery of telehealth and can act as a barrier to uptake. Common problems include poor network access, a lack of technical knowledge or confidence, and limited support availability. Reviews highlighted that access to appropriate, user-friendly equipment and technologies was a key factor in the utilisation of telehealth interventions.

While there is limited evidence to suggest whether a lack of face-to-face interaction significantly impacts on users’ acceptability of telehealth, some evidence pointed to preferences for hybrid delivery models that offer opportunities for face-to-face interaction in addition to telehealth contacts. Blended intervention types, utilising telehealth alongside face-to-face encounters could be a widely acceptable and successful form of intervention delivery. There is recognition that hybridised models provide services with the benefits of extended reach, widened access, and convenience while retaining more personal forms of face-to-face engagement, relationship building, and observation that cannot be achieved digitally.

Telehealth interactions were rated similarly or comparably to in-person care in a number of reviews. However, some findings indicate that rapport and relationship building takes longer to develop virtually compared with face-to-face settings. Given that the FNP service is a home visiting programme based on mentorship and developing a strong therapeutic relationship between nurses and clients, this is an important aspect to consider in terms of clients’ abilities to achieve programme goals and effect positive changes in their lives.^11^ Adopting a hybrid approach, offering telehealth as a supplementary element of care, could help to build a strong positive relationship between clients and providers whilst mitigating some of the barriers posed by virtual interactions.

Some reviews identified that telehealth tools enabled more connection amongst healthcare colleagues and improved the coordination and quality of care for clients. Other studies highlighted that some practitioners expressed preferences for face-to-face interactions, citing frustrations with a lack of, or even hostile communication, occurring via virtual platforms. It is important for service providers to consider offering flexible arrangements for practitioners based on overall levels of satisfaction and their ability to use telehealth technologies remotely or in a professional setting. Staff should be comfortable in their working environment and be able to communicate well with colleagues to provide the best quality of care for service users. Service providers should aim to regularly review virtual and hybrid team-working structures in tandem with in-person working practices to minimise exclusion.

There was consensus among studies that telehealth interventions could afford healthcare providers and service users opportunities for flexibility, autonomy, convenience, and efficiency. However, the findings also suggest that maintaining a level of structure and punctuality when scheduling telehealth appointments could work to ensure higher attendance levels and help to manage staff time more effectively.

Addressing client and practitioner concerns around privacy and confidentiality is highly important when using telehealth. Concerns may be particularly acute for women seeking support for stigmatised issues (e.g. sexual and reproductive health, HIV, and mental health conditions), which could create a sense of unease between a service user and provider.^55,56^ Some studies identified that a practitioner's work environment can negatively influence a service user's level of comfort and desire to disclose personal information during a consultation. Busy office or clinical settings where a client is aware of other people in the background or where there is a perceived lack of privacy can have a significant negative impact on their experience with a healthcare provider. Home-working and virtual communication requirements implemented due to the COVID-19 pandemic may have also elicited similar concerns regarding the risk of occupants in a practitioner's home overhearing sensitive information. Increased use of telehealth may also prevent more vulnerable service users from accessing safe spaces where confidential disclosures can be made to a practitioner. Service users may also hold concerns about communicating sensitive information virtually to their healthcare providers.

Healthcare practitioners should pay close attention to a client's privacy environment and ensure that alternative provisions can be made. Good levels of organisational support should be in place to ensure that rigorous standards of data protection, information governance, and confidentiality can be maintained by staff using telehealth.

At an organisational level, secure, regularly updated software and devices should be available for practitioners and appropriate support channels should be in place for practitioners and service users. It is also recommended that organisations provide appropriate and private on-site spaces for staff to conduct telehealth consultations. Regular training should also be available to ensure that appropriate privacy and confidentiality standards and safe working practices can be maintained in clinical and home-working settings.

Access to training and support for healthcare providers using telehealth are vital factors for successful implementation and delivery and were found to be a key determinant of acceptability. Access to sufficient training in relevant technologies positively impacted staff and client experiences during telehealth consultations. Upskilling staff can also increase capacity and career development opportunities for health professionals, who are likely to experience expedited transformation of traditional healthcare to online settings in the near future.

The review also highlights the delicate interplays between the opportunities that may be afforded by telehealth and the need to consider the complex needs and vulnerabilities present in client populations such as those enrolling into FNP. While there may be unique opportunities to expand service access, telehealth is unlikely to provide a ‘one-size fits all’ solution for all service users, especially those with complex vulnerabilities. Professional judgement therefore should be at the heart of decision-making to ensure that clients are being engaged with in the most appropriate and supportive manner. For some, this may involve increased telehealth interactions that allow flexibility, convenience and control for clients to engage with programmes more autonomously. For others, face-to-face contact may be essential if clients are uncomfortable with virtual engagement, or if there are challenges associated with digital access (e.g. poor digital literacy, limited resources, device access or connectivity issues).

### Limitations

The review has several limitations. However, the rigorous process of multiple reviewers searching, blind screening, and cross-checking is likely to reduce these limitations.

The search strategy sought to capture a breadth of related intervention types and contexts of interest, however, the list of terminology used is not exhaustive. Search limits were put into place, in line with rapid review methodology guidance,^20^ to ensure a manageable volume of results for screening in a timely manner. Due to these constraints, it is possible that a proportion of relevant literature may have been overlooked that would otherwise have been captured by a more thorough systematic review process. For example, the exclusion of studies from low-income countries means that the review has not captured findings from lower-resource and culturally diverse settings that could hold additional key lessons for telehealth delivery across a variety of contexts and population sub-groups. We would encourage researchers undertaking a full traditional systematic review to explore evidence from a range of settings with mixed resource and skill availability to draw transferable lessons and generalisable findings appropriate for diverse global contexts.

The review is also limited by the temporality of the phenomenon. Most reviews included were published prior to the COVID-19 pandemic, and thus may not fully represent the current reality of telehealth implementation in programmes such as the FNP. Findings from older studies may also be limited due to the pace and scale of digital transformation and enhancements that have occurred generally in recent years. Supplementary primary studies relevant to COVID-19 were included to contextualise the findings in recognition of a unique situation, however, inclusion of these studies is far from comprehensive. While rapid implementation of telehealth has taken place in response to COVID-19, we hope many of the retrospective findings presented here will continue to retain contextual relevance for future implementation.

Relevant findings from different intervention types, settings, and populations sharing similarities with home visiting programmes such as FNP have been presented here. Findings therefore may not be fully representative of the current experiences of practitioners and clients specifically involved in FNP as the extent of telehealth utilisation and delivery modes will vary according to various programmes, locations, and client vulnerability. Most included studies presented findings from international contexts out with Scotland and the UK so some cultural and organisational differences may also exist in terms of population needs, challenges, and service provision.

## Conclusions

This rapid review highlights opportunities and disadvantages of incorporating telehealth into early parenthood programmes such as FNP. Notably, the evidence highlights examples of telehealth improving access for key population sub-groups inclusive of young people, pregnant women, first-time mothers, women living with mental health conditions, and women with fewer resources and migrant groups. Acceptance of telehealth in client groups is enhanced when interventions are tailored or personalised towards individuals. For practitioners, acceptance is improved when interventions are user-friendly and accompanied by high levels of training and support.

The overall success of telehealth interventions in home visiting programmes will likely be determined by their design and implementation strategy. Interventions that have a strong grounding in behaviour change theories, such as FNP, are also more likely to yield higher levels of engagement and impact. Hybrid models were also positively regarded, providing a means for the benefits of in-person interactions, such as relationship and rapport building to be realised with those of telehealth (e.g. convenience, flexibility, and reduced travel times).

Technical issues, including limited connectivity and access to devices, comprise some of the biggest barriers to successful uptake. Decision-makers should be mindful of mitigating and minimising digital exclusion amongst service users by providing adequate support, information, and exercising professional judgement around the appropriateness of virtual engagement for various clients.

Maintaining high standards of privacy and confidentiality is of vital consideration when planning to use telehealth. Clients may have complex concerns and practitioners should seek to identify and address these from an early stage. Practitioners should be well supported by strong levels of organisational support and training to ensure that rigorous standards of data protection, privacy, and confidentiality can be adhered to.

These findings are timely in response to the rapid implementation and expansion of telehealth during COVID-19 and it is hoped that these can be taken into consideration to support the implementation and sustainability of telehealth to support families in this context. Further research is required to understand barriers and facilitators specific to telehealth implementation in home visiting programmes and to explore the sustainability and impact of incorporating telehealth more widely into these services.

## Supplemental Material

sj-docx-1-dhj-10.1177_20552076221123711 - Supplemental material for Understanding the use of telehealth in the context of the Family Nurse Partnership and other early years home visiting programmes: A rapid review Click here for additional data file. Supplemental material, sj-docx-1-dhj-10.1177_20552076221123711 for Understanding the use of telehealth in the context of the Family Nurse Partnership and other early years home visiting programmes: A rapid review by Kathleen Morrison, Thomas Hughes and Lawrence Doi in Digital Health

sj-docx-2-dhj-10.1177_20552076221123711 - Supplemental material for Understanding the use of telehealth in the context of the Family Nurse Partnership and other early years home visiting programmes: A rapid review Click here for additional data file. Supplemental material, sj-docx-2-dhj-10.1177_20552076221123711 for Understanding the use of telehealth in the context of the Family Nurse Partnership and other early years home visiting programmes: A rapid review by Kathleen Morrison, Thomas Hughes and Lawrence Doi in Digital Health

sj-docx-3-dhj-10.1177_20552076221123711 - Supplemental material for Understanding the use of telehealth in the context of the Family Nurse Partnership and other early years home visiting programmes: A rapid review Click here for additional data file. Supplemental material, sj-docx-3-dhj-10.1177_20552076221123711 for Understanding the use of telehealth in the context of the Family Nurse Partnership and other early years home visiting programmes: A rapid review by Kathleen Morrison, Thomas Hughes and Lawrence Doi in Digital Health
