# Supplementary material for: Understanding the use of telehealth in the context of the Family Nurse Partnership and other early years home visiting programmes: A rapid review
Source: Digit Health. 2022 Nov 14;8:20552076221123711. doi: 10.1177/20552076221123711 (PMC9666867; doi:10.1177/20552076221123711)
Supplement: sj-docx-1-dhj-10.1177_20552076221123711 - Supplemental material for Understanding the use of telehealth in the context of the Family Nurse Partnership and other early years home visiting programmes: A rapid review [file sj-docx-1-dhj-10.1177_20552076221123711.docx]

**Supplementary file 1. Search strategy**

| SEARCH STRATEGY | | |
| --- | --- | --- |
| # | **Search** | #1 AND #2 AND #3 AND #4 |
|  | **Domain** | **Search** **Terms** |
| #1 | Population | “adult” OR “teenager” OR “teen” OR “family” OR “parent” OR “carer” OR “mother” |
| #2 | Intervention | “telehealth” OR “tele-health” OR “telemedicine” OR “tele-medicine” OR “ehealth” OR “e-health” OR “Telemonitoring” OR “Telecare” OR “telephone” OR “video conferenc*” OR “video call” OR “video consultation” OR “text messag*” OR “SMS messag*” OR “phone call” OR “telenursing” OR "mhealth" OR "technolog*" |
| #3 | Outcomes | “Continuity of care” OR “continuity of carer” OR “relationship” OR “rapport” OR “satisfaction” OR “acceptance” OR “engagement” OR “uptake” OR “quality” OR “mode of delivery” OR ”retention” OR “observation” OR “perceptions” OR “service delivery” OR “delivery of care” |
| #4 | Study Design | “review” OR “meta-analysis” |

**Databases:** Medline, CINAHL, Cochrane Library
