# Supplementary material for: Understanding the use of telehealth in the context of the Family Nurse Partnership and other early years home visiting programmes: A rapid review
Source: Digit Health. 2022 Nov 14;8:20552076221123711. doi: 10.1177/20552076221123711 (PMC9666867; doi:10.1177/20552076221123711)
Supplement: sj-docx-2-dhj-10.1177_20552076221123711 - Supplemental material for Understanding the use of telehealth in the context of the Family Nurse Partnership and other early years home visiting programmes: A rapid review [file sj-docx-2-dhj-10.1177_20552076221123711.docx]

**Supplementary file 2. Main findings table**

**Table 3. Rapid Review Findings**

| Review/Date | Setting | Methods/ Included study designs (number of studies included) | Population | Intervention | Relevant Findings | AMSTAR Score |
| --- | --- | --- | --- | --- | --- | --- |
| Ames et al. 2019. | Sixteen sources in high-income countries: United States, United Kingdom, Canada, Australia.  Nineteen sources in low-middle income countries: Cambodia, Cameroon, Nigeria, Ghana, Kenya, Lesotho, South Africa, Peru, Sierra Leone, and Uganda | Qualitative evidence synthesis    Qualitative studies that: had a qualitative design, were primary studies using qualitative methods, and relied on qualitative analysis. Mixed-methods studies were included.  (N = 35) | Adolescent youth/ adults (ages 10-24) recruited using sexual and reproductive health services | SMS, Voice Calls, Voice Messages, WhatsApp, and Facebook Messenger | Positive impact: Providing support and connectedness to provider and positive impact on the client-patient relationship.  Clients dealing with stigmatised health conditions (e.g. HIV, family planning and abortion) worried about confidentiality and privacy.  Patients wanted messages to be sent from a provider, who was polite and encouraging. They did not want to feel pressured or lectured.  Barriers to participating was a network connection, access to electricity, device usability and issues tied to confidentiality and privacy. Barrier for those who speak a minority language or low digital literacy. | Moderate |
| Bailey et al. 2018. | All sources came from the United States and the United Kingdom. | Scoping Review  (Quality Improvement projects, Discussion papers, clinical audits, and a qualitative study)  (N = 11) | Midwives and women in prenatal care. | Telephone triage: Assessment conducted via telephone call. | Expectations of Midwives using telehealth: be confident, experienced, excellent communication skills, highly-developed clinical decision making skills, be able to maintain confidentiality, and good telephone etiquette.  Findings suggested a lack of formal training in telephone triage.  Challenges of telephone triage: Increased workload, environment (i.e. telephone calls being taken in a public/ private space) could exert pressure on women to give standardised answers due feelings of surveillance., Lack of funding for telephone triage, Inability to see woman, failure to determine the disposition of the caller, challenges to legal responsibility, poor telephone procedures and documentation, and breaches of confidentiality.  Achieving quality: Need for formal training (i.e. around safety, communications and decision-making skills, determining the acuity and disposition of the women), necessary documents (i.e. policies, guidelines and proforma), a private environment to provide confidentiality and comfort, and having time to conduct the triage. | Low |
| Brewster et al. 2014. | UK, Canada, the Netherlands, Australia and Denmark. | Systematic Review of Qualitative and Quantitative evidence.  (Quantitative surveys, mixed-methods studies, and qualitative studies)  (N = 14) | Frontline Healthcare Staff | Telehealth to monitor people’s health in their own home.  Response delivered by staff by telephone call, videoconferencing or face-to-face delivery. | Staff acceptance and awareness is key to the implementation of telehealth.  Staff need to be able to feel that they can use their clinical knowledge and judgement in patient management via telehealth.  Reliability and accuracy of the telehealth equipment is important for staff acceptance.  Equipment needs to be easy to use for to staff to feel confidence to introduce it to patients. | Low |
| Cunningham et al. 2021 | United States | Narrative Review  (N = 55) | Children living with mental conditions and their parents | Telehealth for paediatric mental health nursing during COVID-19 | Access to telehealth was facilitated when health insurance companies were willing to cover telehealth services, where there was sufficient access to resources, and relaxed regulation around the use of technologies.    In some instances, telehealth helped to increase access to care for patient who previously were faced with in-person barriers.    It may not be suitable for certain services (e.g. treating complex psychopathology, and certain assessments).    Safety concerns, barriers to internet/technology access. | Low |
| Endler et al. 2019. | Netherlands, Australia, Canada, and the United States | Systematic Review of Qualitative and Quantitative evidence.  Included study designs: Retrospective cohort, prospective cohort, descriptive and qualitative studies.  (N = 15) | Women using medical abortion services via telemedicine | Telephone and Video Calls | Higher acceptability of telehealth delivered services among patients was due to reduced travel and waiting times.  Higher acceptability was also recorded among providers.  Telehealth delivery offered greater flexibility, better distribution of resources, fewer cancellations and delays, and reduced travel times to the clinic.  Minimal impact on the client flow.  Minimal impact on patient-provider relationship. | Moderate |
| Gentles et al. 2010. | 17 different countries: United States, Canada, Australia, Greece, Japan, Israel, Taiwan, Netherlands, Norway, Germany, Spain, France, Ireland, and Multiple countries. | Scoping review (Qualitative, mixed methods studies, quantitative [RCTs, non-RCTs, descriptive studies, and before-and-after studies])  (N = 112) | Patient or caregiver of a paediatric patient (30 different conditions e.g. asthma, type 1 diabetes special needs and psychiatric disorders). | Health Information Technologies: Internet, Intranet, Telephone calls, videoconference, email, SMS, and manual download. | Establishing continuity of care: HITs were seen to extend care to patients in the community beyond the setting where they traditionally access care (e.g. hospitals)  Addressing time constraints: There was an increase efficiency of care or reducing time burden on health care providers.  Bridging geographical boundaries: reducing the need for patients to travel or providing access to distant specialists. | Low |
| Gonçalves-Bradley et al. 2020. | Trials mainly based in North America and Europe. The remainder of sources coming from the Dominican Republic, Turkey, Uganda, and Mongolia. | Systematic Review of Randomised Control Trials (RCTs)  (N = 19) | Healthcare providers providing patient care through telemedicine | Care delivered through Mobile Devices | The trials revealed a reduction in the time between the presentation and management of the health problem.  Could reduce referrals and clinic visits for people suffering from skin conditions and chronic kidney disease. And an increase in the likelihood of receiving an eye examination for people with diabetes.  Little difference in patient reported quality of life outcomes or to the provider’s satisfaction and acceptability, or cost.  Most trials did not measure any technical problems. One trial did report mobile phones not being charged or being lost. | High |
| Hanach et al., 2020 | Netherlands | Systematic Review and Meta-Analysis  (N = 10) | Postnatal women with no previous experience of mental disorders | Telemedicine interventions (e.g., telephone support, social media, mobile apps and websites) delivered during the postnatal period to improve postpartum depression symptomology in women with no prior experience of mental health disorders. | Women who were part of the intervention group reported significant improvements in postpartum depression.    The completion rates were 80%, compared with 76% for the control group.    Patients seemed to be highly satisfied. | Moderate |
| Harris et al., 2020 | Canada | Systematic Review & Meta-Analysis  (N = 9) | Families with at least one of the following social disadvantages: low socioeconomic status (i.e., low-income listed as inclusion criterion or recruitment from specific community agency programmes designed to service low-income families), single parenthood, and/or young parenthood (maternal age at birth of target child < 25 years) | Interventions aimed at improving parenting behaviour, and/or parental psychological well-being with the intervention, or a component thereof, delivered by computer, online/internet mode, cell phone, smartphone, tablet, or video/DVD. | Interventions incorporating direct contact (i.e., in-person, video conferencing or phone calls) produced greater positive change in parental psychological well-being (g = .68) than interventions without contact (g = −.02).    The few studies that compared the effectiveness of technology-assisted interventions with in-person programs showed greater effects for face-to-face interventions, relative to technological strategies.    Length of intervention also proved a significant moderator; shorter interventions produced greater positive change. | Low |
| Lattie et al. 2019. | The majority of studies took place in United States. Other studies were based in the UK, Ireland, Canada, Australia and China. | Systematic Review  (N = 89) | University students | Digital mental health platforms available via mobile phones, websites, virtual reality systems and offline computer programmes | Usability and acceptability: generally favourable. Low response rates.  Adoption and uptake: High rates of attrition and low rates of sustained programme use. | Moderate |
| Lavender et al. 2013 | United States, Canada, Australia, Thailand, New Zealand, Italy, Zanzibar and Scotland. | Systematic Review (RCTs)  (N = 29) | Pregnant and postnatal (the first six weeks after birth) women | Telephone calls delivered by healthcare workers, peer support workers, or using automated messaging services. | Higher rates of maternal satisfaction found in studies among women who received telephone services than the control groups.  There was no difference between the women who received the telephone service and those who didn’t in terms of healthcare utilisation and maternal anxiety. | High |
| Le Blanc et al., 2020 | Canada and Australia | Scoping Review  (N = 69) | Patients and providers in rural settings (Australia and Canada) | eHealth interventions (video conference calls, phone calls and digital monitoring services) to support health needs in rural communities | Positive aspects of eHealth:   - Decreased travel time - Cost effective - Increased access to care.     Most common problems with eHealth:   - Technological problems - Lack of face-to-face contact - Limited training - Resource disparities (lack of high-speed internet in rural areas)     Patients reported a number of benefits: less disruption to family life, reduced anxiety, and improved recovery time.    Providers noted: increased connection with colleagues, improved support for complex care and greater eLearning opportunities. | Low |
| Nordtug et al. 2018. | United States, Australia, Sweden, and Spain. | Systematic Review (two RCTs and six pilot studies [two qualitative, two mixed-methods, and two quantitative]).  (N = 8) | Nurses for people of all ages, both sexes, and with chronic illnesses or need of nursing follow-up. | Videoconferencing | Access and Equipment: Network access and quality of the video call influences the success of the intervention.  Training and use: Need for sufficient information and training before the use of VC.  Contact time: Some of the studies allowed for only the patient to initiate the VC. Some studies allowed for contact during anytime of the day and night, others restricted contact to only the evenings. Some had fixed time session, whilst others allowed for ad hoc possibilities. Overall, there was a need to structure VC session with regard to who initiates contact, when, how often, and where.  Privacy: Nurses tried to preserve patients’ privacy by ensuring that others were not listening or seeing the VC. Patients had more control over where the appointment took place, e.g. their homes, meaning there was little threat to their privacy.  Close Collaboration: VC brought a deeper dimension than telephone calls. Seeing faces and body language made it easier for nurses to understand the information the patient needed.  Assessment and follow-up: VC was suitable in follow up of medical health problems, information provision, and discussion of patients’ and families’ emotions, needs, and wishes. VC provided families with more flexibility, as they did not need to travel to a hospital. | Low |
| Odendaal et al. 2020. | Eleven studies from high income countries (Australia, Canada, United States, Scotland, Norway, and the Republic of Ireland.  Thirty two studies from low-middle income countries (India, Bangladesh, Brazil, Ethiopia, Ghana, Malawi, Kenya, Lesotho, Rwanda, South Africa, Swaziland, Tanzania, Timor-Leste, Myanmar and Uganda) | Qualitative evidence synthesis (qualitative studies)  (N = 43) | All categories of healthcare workers involved in providing primary care services to clients. | mHealth technologies (the use of mobile devices to create, store, retrieve, and transmit data in Realtime between users, and interventions used by health workers to provide support to clients). | Connection with Colleagues: mHealth was seen to improve connection between colleagues, and thus thought to improve the coordination and quality of care.  mHealth offered health workers the ability to take on new tasks, work flexibly, and reach clients in hard-to-reach areas.  Relationship: Overall, health workers felt that mHealth helped to improve the care and relationship with clients. But they felt that some clients needed face-to-face contact.  Health workers understood the importance of protecting confidentiality when using mHealth technologies.  Perceptions and use of mHealth was tied to the cost and poor network access. Some HWs did not mind covering the extra cost, others complained that the phone credit was not delivered on-time.  Being accustomed to using mobile phones was positively correlation with more acceptance of mHealth. | Moderate |
| Poorman et al. 2015. | N/A | Systematic review (RCTs, non-randomised controlled trials, cohort studies, uncontrolled trials, cross sectional and ecological studies, and pilot studies)  (N = 48) | Women of reproductive age (12-50 years old) and infants under the age of 2 | Text messaging intervention in the preconception, prenatal or postpartum period | Interventions should follow established theories of behaviour change in text messaging interventions, as much as in other behavioural change interventions.  The outcomes of text messaging interventions need to be closely aligned with the content, especially for comprehensive pregnancy programmes.  The use of text messages to promote maternal health has tremendous potential to reach pregnant women and new mothers, particularly those with few resources. | Low |
| Seko et al. 2014. | Australia, Republic of Ireland, Italy, United States, and New Zealand. | Scoping review (15 journal articles [RCTs, quasi-experimental studies, pilot studies quantitative and qualitative], 1 book chapter, and 1 conference proceeding)  (N = 17) | Young people (aged 13-24 years old using mobile mental health service) | Mobile mental health services (SMS, phone and/ or apps) | Privacy: Phone calls provided users with sufficient privacy to support therapeutic practices. The use of mobile phones offered users personal space allowing for increasing sense of control over privacy. Being able to ensure privacy and confidentiality was the most common concern.  Flexibility: Young people appreciated the flexibility offered by mHealth. They preferred SMS and apps over telephone and video calls as it felt less intrusive and gave them the opportunity to do it in their own time.  Accessibility/Usability: Large-scale ownership of mobile phones was advantageous for facilitating large-scale use. Mobile phone health was considered more user-friendly less of a need for training. | Low |
| Verhoeks et al. 2019. | United States, the United Kingdom, the Netherlands, Sweden, Norway, Australia, Canada, and New Zealand. | Systematic Review: Qualitative, Quantitative and Mixed-methods sources.  (N = 16) | All women receiving telehealth services | Telehealth (i.e. texted based online counselling via email or chat, or internet delivered self-help programmes) | Lowers the threshold: women (based on their experiences and expectations) were able to seek healthcare more easily.  Anonymity: Women had greater anonymity through the internet making it easier for them to talk about their problems. Some felt that talking about their thoughts, feelings, and difficulties should be done in person.  Women expected to struggle to develop a therapeutic relationship with their provider over telehealth. However, after using it some felt that the relationship was the same as with face-to-face contact. Some still felt that relationship was less close and personal.  Flexibility: Women were attracted to the flexibility of e-health, as it gave them the ability to do things at their own time, place and peace. Flexibility, however, was seen to be a barrier in completing the treatment, because of reduced feelings of obligation and motivation. | Moderate |
| Walsh et al. 2016. | Most of the papers were from the United States or Europe, with the remaining studies coming from Australia, Japan and an international study report. | Systematic review (Quantitative findings on participations rates, and qualitative findings on patient views).  (N = 42) | Patients suffering from adjustment disorders, eating disorders, anxiety disorders, mood disorders, schizophrenia and other psychotic disorders. | Technology used to monitor subjectively symptoms, emotions and behaviours outside of routine clinical appointments, that provide feedback to clients, providers and/ or other parties (such as a carer) | Overall high levels of acceptance were reported with moderate to strong participations rates.  Diagnosis (i.e. the type of mental health condition) may influence the experience of the participants.  The lack of prompting and guidance may contribute to reports that there was a lack of support. | Low |
