# Supplementary material for: Understanding the use of telehealth in the context of the Family Nurse Partnership and other early years home visiting programmes: A rapid review
Source: Digit Health. 2022 Nov 14;8:20552076221123711. doi: 10.1177/20552076221123711 (PMC9666867; doi:10.1177/20552076221123711)
Supplement: sj-docx-3-dhj-10.1177_20552076221123711 - Supplemental material for Understanding the use of telehealth in the context of the Family Nurse Partnership and other early years home visiting programmes: A rapid review [file sj-docx-3-dhj-10.1177_20552076221123711.docx]

**Supplementary file 3. Supplementary evidence table**

| Author, year, location, source type | Title | Aim | Population | Intervention | Comparator Face to face delivery/control | Relevant Findings |
| --- | --- | --- | --- | --- | --- | --- |
| Hall and Bierman, 2015, United States, Review | Technology-assisted interventions for parents of young children: Emerging practices, current research, and future directions | Study reviews existing publications that describe the application and evaluation of technology-assisted interventions for parents of young children. | Parents of young children under the age of 5 | Telehealth technology: video consultation, internet, web-based education, and telephone | Face-to-face delivery | Feasibility and acceptability:   - Web-based platform for information: internet uses for information was high across all groups, but social class impacts their receptiveness. Higher socioeconomic status (SES) families more likely to engage with this. - Online discussion forums/blogs to promote social support: effective way to link parents with each other. Format less attractive to low SES families. - Mobile devices and videoconferencing: Reduced cost (compared with home visiting) and mixed satisfaction with the technology across groups.   Impact:   - Web-based interventions: Can effectively promote improvements in parent knowledge and feelings of parent efficacy, as in some case reduce parenting stress. - Videoconferencing: Delivered educational materials and providing modelling and guidance to parent’s online produces gains in parent-reported knowledge, attitudes, and (sometimes) behaviours relative to the comparison group who did not use videoconferencing. |
| Smith et al., 2020, Australia, Letter to the Editor | The role of telehealth in supporting mothers and children during the COVID-19 Pandemic | Perspectives on the Communicating Healthy Beginnings Advice by Telephone (CHAT) intervention during the COVID-19 pandemic. | Mothers of young infants using the CHAT telehealth service | Telephone calls and SMS | Face to face delivery | - Participants reported high satisfaction with telephone call and the information support delivered reported among women. - Intervention had the ability to engage harder-to-reach population groups (e.g.. socially isolated women in regional areas, low SES families, women who speak English as a second language, and women dealing with mental health issues). - Service offered convenience to mother (able to call at any time she wished). No reliance on the availability of transport, location, or weather conditions. Telephone calls were also offered after hours and on the weekend. - Called for the need to integrate telehealth into the existing health system. |
| Traube et al., 2020, United States, Pilot Feasibility Study | Advancing Home-based parenting programs through the use of telehealth technology | Article describes a pilot feasibility study focusing on model fidelity for delivering a home-based parenting program via a university-based telehealth interactive video conferencing technology. | New parents of children over the age of three (large representation of Hispanic families) | Videoconferencing technology | Face to face delivery | Fidelity:   - Families participated for an average of 5 months, despite being offered up to 24 months of service. - Most families leaving the programme were discharged due to missing multiple visits or being unresponsive to reengagement attempts. No families requested to leave the program due dissatisfaction or because they were moving, which is commonly seen in on-ground programs.   Parental Satisfaction:   - Parental satisfaction was very high with 80% saying they were “very satisfied” with the telehealth program. - Relationships and rapport with their practitioner was positively rated with 90% of participants “strongly agreeing” that they felt comfortable speaking with their practitioner using telehealth and all participants reported feeling that their practitioner was “genuinely interested in my family” and able to identify their family’s strengths. - Eighty-nine percent of participants said their experience was the same or better than in-person programs in which they had participated. |
| CDC, 2020, United States, Report | Using Telehealth to expand access to essential health services during the COVID-19 Pandemic. Using telehealth services | To describe the landscape of telehealth services and provide considerations for healthcare systems, practices, and providers using telehealth services to provide virtual care during and beyond the COVID-19 pandemic. | No specific population | Telehealth modalities:  Synchronous: telephone call or video conference using smart phone, computer, or tablet.  Asynchronous: Areas where messages, images or data are collected at one point, and interpreted or responded to later.  Remote patient monitoring: Direct transmission of a patient’s clinical data from a distance to healthcare provider. | Face-to-face delivery | Benefits and potential uses of telehealth:   - Provides low-risk urgent care for non-COVID-19 conditions, identify those persons who may need additional medical consultation or assessment, and refer as appropriate - Engage in case management for patients who have difficulty accessing care (e.g., those who live in very rural settings, older adults, those with limited mobility) - Provide education and training for healthcare practitioners through peer-to-peer professional medical consultations that are not locally available, particularly in remote areas.   Strategies to increase the uptake of telehealth:   - Provide outreach to patients with limited technology and connectivity and offer flexibility in platforms that can be used for video consultation, or non-video options, when possible - Promote and optimise the use of telehealth services for the safety of healthcare workers and patients from COIVD-19 risk.   Limitations of telehealth:   - Situations in which in-person visits are more appropriate due to urgency, underlying health conditions, or inability to perform adequate observations - The need to address sensitive topics, especially if there is patient discomfort or concern for privacy - Limited access to technological devices (e.g., smartphone, tablet, computer) needed for a telehealth visit or connectivity issues - Level of comfort with technology for practitioner and patients - Cultural acceptance of conducting virtual visits in lieu of in-person visits by practitioner and patients |
| Owen et al., 2020, Australia, Feasibility study | Feasibility and acceptability of using telehealth for early intervention parent counselling | This study aimed to evaluate clinicians’ and parents’ experience of accessing videoconference (VC) counselling within child health services. | Parents or caregivers of young children (0–8 years)  Clinicians (Early intervention parenting clinicians) | Synchronised video conferencing: Subsequently, launched a Telehealth Portal where parents with smartphones, tablets or computers and adequate internet access could connect directly with a practitioner in a collaborative meeting room. | In-person or telephone session | - The results indicate that VC is a viable alternative for some families, especially for those that would not be able to access in-person counselling. - Most parents were satisfied with the VC counselling, despite mild technical issues, and improved in their efficacy and confidence in parenting. - Parents reported a preference for a flexible hybrid mode, combining in-person counselling with the convenience of VC. - Parents were interested in VC appointments as it accommodated reduced travel time, work commitments, health issues and competing time demands. - Upskilling clinicians using interactive VC peer sessions resulted in increased skills, knowledge, and confidence in using VC for counselling. - Some clinicians felt that it took longer to build rapport with clients. - The study demonstrated that providing professionals with VC equipment, knowledge, and practice, effectively supported the commencement of telehealth practice. |
